# 中国控制吸烟协会吸烟与疾病控制专业委员会成立大会纪要及倡议书

**DOI:** 10.3779/j.issn.1009-3419.2010.07.17

**Published:** 2010-07-20

**Authors:** 中国控制吸烟协会吸烟与疾病控制专业委员会肿瘤学组

## 1 中国控制吸烟协会吸烟与疾病控制专业委员会成立大会纪要

中国控制吸烟协会吸烟与疾病控制专业委员会成立大会暨新闻发布会于2010年5月25日在北京大学人民医院多功能厅举行，来自全国各地近百名临床专家出席了成立大会。成立大会由中国控制吸烟协会常务理事、首都医科大学肺癌诊疗中心主任支修益教授主持，中国控制吸烟协会许桂华常务副会长、杨功焕副会长、胡大一副会长、段佳丽副秘书长以及北京大学人民医院院长王杉教授在成立大会上致辞，许桂华常务副会长宣读了民政部对中国控制吸烟协会吸烟与疾病控制专业委员会成立申请的批复，胡大一副会长宣读了卫生部领导的贺词。中国控制吸烟协会杨功焕副会长主持了中国控制吸烟协会吸烟与疾病控制专业委员会主任委员和副主任委员的选举工作。经过民主选举，北京大学人民医院胡大一教授全票当选为中国控制吸烟协会吸烟与疾病控制专业委员会主任委员，首都医科大学宣武医院支修益教授、卫生部中日友好医院林江涛教授、中国疾控中心汪宏梅教授当选为副主任委员。

胡大一教授在成立大会上指出：我国的控烟形势不容乐观，作为临床医生，应责无旁贷地担负起引导全社会控烟的义务。林江涛教授代表中国控制吸烟协会吸烟与疾病控制专业委员会宣读了“促进中国控烟行动、做控烟表率”的倡议书。与会领导和专家在倡议书签字。

成立会议后肿瘤学组、呼吸疾病学组、妇幼学组、心血管疾病学组分别召开了第一届常委工作会议，商讨新一届委员会工作计划。

肿瘤学组工作会议总结如下：

**1** 讨论通过吸烟与疾病控制专业委员肿瘤学组组长：支修益、高文、何建行、周清华、石远凯。

**2** 肿瘤学组共有36名委员，建议各位委员安排一名委员助理协助肿瘤学组工作开展，整理肿瘤学组委员及助理通讯录。

**3** 肿瘤学组近期工作计划：

① 5月31日世界无烟日主题是“性别与烟草——抵制针对女性的市场营销”，建议各位常委及委员根据各专业和单位情况开展一次控烟健康教育科普活动。活动后将活动纪要和图片发送给学组秘书王鑫，统一上报总会。

② 开展吸烟与疾病相关因素统计和研究工作：如吸烟与肺癌相关性、了解医生的吸烟状况等，建立中国肿瘤领域吸烟与疾病相关的流行病学资料。

③ 联合搜狐网健康频道、人民网卫生频道和相关媒体，加强控烟宣传及健康教育。

④ 加强组织建设，建立常委例会制度，编辑肿瘤学组内部通讯。

⑤ 承担总会工作、总会网站和通讯投稿，参加总会年会等。

⑥ 建立专项基金，同抗癌医药公司、戒烟药物公司和国际公司合作。

⑦ 重视国内外交流与合作，国内学会协会应与国际控烟机构合作。

## 2 中国控制吸烟协会吸烟与疾病控制专业委员会成立大会倡议书——促进中国控烟行动、做控烟表率

中国是烟草种植、生产、销售大国，也是受烟草毒害最为严重的国家。我国吸烟者总数超过3.5亿，遭受被动吸烟危害的人数高达5.4亿，其中15岁以下儿童有1.8亿。吸烟是心脑血管疾病、癌症、慢性阻塞性肺病等多种疾患的行为危险因素，严重危害着人民健康。2005年我国直接归因于烟草使用所致的肿瘤、心脑血管疾病、慢性呼吸系统疾病等的死亡人数达到120万，且呈现持续增长，预计到2030年将达到200万。

我们来自临床防治一线，深切感受到烟草对健康和生命的威胁；强烈意识到，如不积极应对，烟草将夺去更多生命，影响国家的稳定、发展以及和谐社会的实现。

烟草危害可预可控。我们认为，应该建立政府主导、多方合作、媒体宣传动员和公众广泛参与的机制，全面控烟。为此，我们呼吁：

**1** 认真履行《世界卫生组织烟草控制框架公约》，积极推进控烟相关的各项立法。

**2** 加大对控烟事业，特别是对控烟宣传教育、社区防治示范和干预的投入。

**3** 大众传播媒介与烟草相关疾病防治机构及专家密切合作，积极提供烟草危害防治的宣传服务。

**4** 医疗卫生机构及广大医务人员行动上率先垂范，做控烟先锋，积极推动无烟医疗卫生机构建设，执行对接诊患者常规指导戒烟的首诊负责制，开展烟草危害和控烟的宣传教育。

**5** 积极实施和推广戒烟干预临床诊疗规范，提高戒烟成功率。

**6** 倡导健康生活方式：“戒烟限酒，合理饮食，科学运动，心态平衡”。

我国控烟工作任重道远。让我们行动起来，为民族的健康、国家的昌盛，拒绝烟草，关爱生命，打赢一场控制烟草的人民战争！

